# Global Burden of Disease Estimates of Low Back Pain: Time to Consider and Assess Certainty?

**DOI:** 10.3389/ijph.2024.1606557

**Published:** 2024-02-14

**Authors:** Javier Muñoz Laguna

**Affiliations:** ^1^ EBPI-UWZH Musculoskeletal Epidemiology Research Group, University of Zurich and Balgrist University Hospital, Zurich, Switzerland; ^2^ Epidemiology, Biostatistics and Prevention Institute (EBPI), University of Zurich, Zurich, Switzerland; ^3^ University Spine Centre Zurich (UWZH), Balgrist University Hospital and University of Zurich, Zurich, Switzerland

**Keywords:** global burden of disease, low back pain, GRADE approach, GRADE, disease burden

**The IJPH series “Young Researcher Editorial” is a training project of the Swiss School of Public Health.**

“Low back pain (LBP) is the leading cause of disability worldwide.” Conclusive statements like this are common and usually derive from one of the Global Burden of Diseases, Injuries, and Risk Factors (GBD) studies [1]. The GBD 2021 LBP study [2] generated attention because it included both modelled burden of disease estimates for that year, and global projections to 2050. After accounting for population ageing and growth, this GBD LBP study claims that “more than 800 million people will have low back pain by 2050” [2].

Though GBD LBP modelled estimates are respected by public health researchers, who often include them in their paper introductions, these estimates have limitations that are often overlooked [3, 4]. Before presenting them as evidence, researchers and decision-makers should consider and assess the methodological certainty of GBD modelled estimates of LBP [5, 6]. To make such an assessment, they can use the systematic and transparent framework of the Grading of Recommendations Assessment, Development, and Evaluation (GRADE) guidelines 30 [5], which describes four key dimensions.

To evaluate the certainty of GBD LBP modelled burden of disease estimates, researchers should first consider the certainty of evidence in each of the studies that underpins the model, also known as model inputs. In the GRADE guidelines 30 framework, certainty of model inputs is captured by the **risk of bias** dimension [5]. GBD LBP modelled burden of disease estimates are informed by different types of input: epidemiological studies, opportunistic surveys, and insurance claims data [1]. After mapping out the location or period of interest for these LBP inputs, researchers can assess their risk of bias with existing tools [7]. They can check whether model inputs use validated LBP case definitions, if their instruments are adequate to measure prevalence, and other important characteristics [7]. GRADE 30 explains that the “overall rating of certainty of evidence across all model inputs should be limited by the lowest certainty rating for any input data to which the model outputs have been found sensitive” [5]. Unfortunately, GBD models are intricate and it is challenging to determine how much influence is exerted by individual model inputs in GBD LBP modelled burden of disease estimates. But if researchers and decision-makers carefully consider the **risk of bias** dimension, they can identify the “best-possible” LBP inputs for GBD and determine how to raise the quality of epidemiological and public health research.

A natural next step in assessing certainty of GBD LBP modelled estimates is evaluating unexplained variability in model outputs—the GRADE 30 **inconsistency** dimension [5]. When researchers can offer no plausible explanation for changes in GBD LBP model outputs over multi-year periods, end users should seek to determine the effect this inconsistency may have on health decisions. If researchers and decision-makers thoughtfully apply the GRADE 30 **inconsistency** dimension, their interpretation of modelled estimates across location-years should become more nuanced.

Since GBD has challenges obtaining primary country-level data for LBP [1, 2], researchers need to know how indirect their input data are, compared to the ideal; this is the GRADE 30 **indirectness** dimension. This dimension is especially important when no LBP inputs are available for national and subnational territories because, in this case, modelled and projected estimates could be overly influenced by inputs from neighbouring or higher-income locations. Analysing GRADE 30 **indirectness** should help researchers and decision-makers appreciate model subtleties and encourage them to consider which direct inputs are key for a particular territory.

Certainty of modelled GBD LBP estimates should also be assessed, based on the width of modelled 95% uncertainty intervals around point estimates. This is the GRADE 30 **imprecision** dimension [5]. In health decision making, certainty judgements can vary if 95% uncertainty intervals are different widths. The **imprecision** dimension of GRADE 30 instructs researchers to downgrade the certainty of evidence when outputs are estimated imprecisely for a given health decision—which may be the case in GBD modelled burden of disease estimates of LBP.

In summary, public health researchers and health decision-makers should consider and assess the methodological certainty of GBD modelled estimates of LBP, before using them as conclusive evidence. GRADE 30 offers a systematic and transparent framework for this purpose, and holds promise to improve GBD modelling methods and quality of LBP and public health recommendations globally.
